# Nature‐based coagulants for drinking water treatment: An ecotoxicological overview

**DOI:** 10.1002/wer.10782

**Published:** 2022-08-27

**Authors:** Widad El Bouaidi, Giovanni Libralato, Zakaria Tazart, Ghizlane Enaime, Mountasser Douma, Abdelaziz Ounas, Abdelrani Yaacoubi, Giusy Lofrano, Maurizio Carotenuto, Lorenzo Saviano, Antonietta Siciliano, Vincenzo Romano Spica, Marco Guida, Mohammed Loudiki

**Affiliations:** ^1^ Laboratory of Water, Biodiversity and Climate Change; Phycology, Biotechnology and Environmental Toxicology Research Unit, Faculty of Sciences Semlalia, Department of Biology Cadi Ayyad University Marrakesh Morocco; ^2^ Department of Biology University of Naples Federico II, Complesso Universitario di Monte Sant'Angelo Naples Italy; ^3^ Institute of Urban Water Management and Environmental Engineering Ruhr‐Universität Bochum Bochum Germany; ^4^ Polydisciplinary Faculty of Khouribga (FPK) Sultan Moulay Slimane University Khouribga Morocco; ^5^ Laboratory of Applied Organic Chemistry, Faculty of Sciences Semlalia, Department of Chemistry Cadi Ayyad University Marrakesh Morocco; ^6^ Department of Movement, Human and Health Sciences University of Rome Foro Italico Rome Italy; ^7^ Department of Chemistry and Biology “A. Zambelli” University of Salerno Fisciano (SA) Italy

**Keywords:** coagulation process, ecotoxicology tests, *Microcystis aeruginosa*, plant‐based coagulants, screening study

## Abstract

The intensive human activities extensively contaminated water sources making its treatment a problem of paramount importance, especially with the increasing of global population and water scarcity. The application of natural coagulants has become a promising and environmentally friendly alternative to conventional ones. This study was aimed at evaluating the efficiency of four plant extracts namely *Agave americana* , *Carpobrotus acinaciformis* , *Austrocylindropuntia subulate*, and *Senicio anteuphorbium* as natural coagulants to remove *Microcystis aeruginosa* cyanobacterium from water. The effects of pH (4, 5, 6, 7, 8 9, and 10) and coagulant dose (5, 10, 15, 20, 25, and 30 mg/L) on the coagulation efficiency were investigated. Results showed that plant‐based extracts exhibited high coagulant abilities significantly contributing to the removal of *M. aeruginosa* cells up to 80% on a case‐by‐case basis. The ecotoxicity ( *Daphnia magna* , *Aliivibrio fischeri* , *Raphidocelis subcapitata*, and *Sorghum saccharatum* ) was absent or presented very slight acute toxicity up to 12.5 mg/L being *S. anteuphorbium* the least toxic.

**Practitioner Points:**

- Nature‐based plant extracts showed removal rates up to 80%.
- Lower pH and *A. subulate* and *S. anteuphorbium* were the most efficient coagulants
- Toxicity effects were plant extracts‐based and dose function.
- *A. subulate* and *S. anteuphorbium* were the least toxic extracts.

## Graphical abstract

Nature‐based coagulants can provide low impact solutions to *Microcystis aeruginosa* reduction from drinking water treatment.

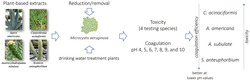

## INTRODUCTION

Water resources devoted to drinking activities are experiencing worldwide serious concerns due to eutrophication and especially toxic cyanobacterial blooms (CyanoHABs) (Paerl et al., 2014). High concentrations of these organisms in raw water may lead to several problems such as the release of toxic secondary metabolites, taste and odor compounds, and disinfection by‐product precursors (Clemente et al., 2020; El Bouaidi et al., 2022). The prevalence and distribution of toxic CyanoHABs in freshwater is increasing, making their removal one of the major challenges of drinking water treatment plants (DWTPs) (Anderson et al., 2002; Hudnell, 2008; Lopez et al., 2008). To address this alarming state, various water treatment technologies have been pursued to ensure adequate supplies of water and to meet water quality standards (Ang & Mohammad, 2020). These technologies fall into three main categories, namely, physical (settling and membrane filtration), chemical (coagulation, electrochemical, ion‐exchange, oxidation, and disinfection), and biological treatment (phytoremediation, microbial biodegradation, digester, and bioreactor) processes (Hamzah et al., 2017; Kumar & Quaff, 2018; Nimesha et al., 2022; Tan et al., 2018).

Coagulation‐flocculation (C/F) process represents one of the most efficient treatment methods to remove CyanoHABs in DWTPs (Vieira et al., 2010). It is considered as a crucial stage in which the first step is carried out to remove the suspended impurities such that the treated water is of desirable quality for subsequent purification processes (Jiang, 2015). C/F is being considered as one of the simple and cost‐effective approaches that can efficiently remove suspended impurities in water (Ang & Mohammad, 2020; Jiang, 2015). C/F is firstly performed by adding chemical coagulants, usually based on aluminum or ferric salts, which promotes the agglomeration of particles in the form of flocs (Betatache et al., 2014; Chen et al., 2013; Ghosh et al., 1994; Shin et al., 2008), followed by the addition of chemical flocculants, increasing the floc density and promoting the formation of particles with larger dimensions. The formed flocs will be subsequently settled or floated according to their density, whereas the unflocculated particles or less‐dense flocs are removed by filtration (Yao et al., 1971).

Despite the high performance of the conventional coagulants and flocculants used for water purification, their residuals in treated water (e.g., aluminum) are suspected to be linked with neurodegenerative diseases such as Alzheimer's, as well as neurotoxic and carcinogenic effects (Camacho et al., 2017).

It has been reported that some commonly used coagulants and flocculants produce an enormous amount of non‐biodegradable sludge, which need to be properly managed (Betatache et al., 2014; Bondy & Campbell, 2018; Jodi et al., 2012). Moreover, an adjustment of the pH by the addition of high amounts of chemicals is sometimes required to sanitize high turbid water, which further increases the operational cost of the whole process.

Recently, researchers focused their effort to develop new eco‐friendly substances for water purification as alternatives to conventional chemicals (Camacho et al., 2017; Teixeira et al., 2017). Natural coagulants present several advantages. In addition to their relative low cost (El Bouaidi et al., 2022), natural coagulants allow less sludge production, and the produced sludge is bio‐degradable and contains no harmful substances, which allow their further valorization in agronomic application (Hameed et al., 2016; Kansal & Kumari, 2014).

Seeds of *Moringa oleifera* are among the most studied plant seeds that have been proven to be effective in removing turbidity from water (Vunain et al., 2019). However, less research investigations have been devoted to evaluating the effectiveness of plant‐based coagulants to remove CyanoHABs from water sources. According to Teixeira et al. (2017), extracts from *M. oleifera* seeds can remove *Microcystis aeruginosa* cells with an efficiency of about 80%. In a previous study, El Bouaidi et al. (2020) have evaluated the potential of *Vicia faba* seeds and *Opuntia ficus indica* cladodes to mitigate CyanoHABs blooms. The results showed that the two plant‐based coagulants were able to remove up to 85% of *M. aeruginosa* cells with a coagulant dose of 5 and 10 mg/L. These results provide sufficient evidence that natural coagulants derived from locally plants could be used as alternative to conventional ones to remove CyanoHABs blooms from water sources. C/F is certainly affected by several parameters including pH, coagulant dose, initial turbidity of water, temperature, and the origin and the nature of the used coagulant (Freitas et al., 2015; Gottfried et al., 2008; Kukić et al., 2015; Sher et al., 2013).

In this study, several toxicity tests have been performed to evaluate the potential toxicity of four plant‐based coagulants, namely, *Carpobrotus acinaciformis*, *Agave americana*, *Austrocylindropuntia subulate*, and *Senecio anteuphorbium*, to ensure the safety of the treated waters after coagulation treatment to remove CyanoHABs from water sources.

*C. acinaciformis* belongs to Aizoaceae family, which is mainly found in Mediterranean climate regions (Campoy et al., 2018), and are among the most widespread invasive plants along the Mediterranean coasts (Jucker et al., 2013; Novoa et al., 2013). This species is a succulent perennial herb (Campoy et al., 2018). *A. americana* is a monocotyledonae plant belonging to Agavaceae family and is widespread in the tropical and subtropical regions. It is characterized by the presence of fibers that are among the strongest, most resistant to thermal and dryness and the stiffest (Hulle et al., 2015). Even though it is considered as an offensive plant in southern parts of Africa, *A. americana* is widely cultivated for ornamental purposes owing to its pale yellow‐margined leaves and ease of reproduction and cultivation (De La Torre et al., 2018; Niechayev et al., 2019). *A. subulate* is a succulent plant belonging to family of Opuntioideae and considered as the largest in opuntias (Boke, 1980; Britton & Rose, 1919). This group has been widely used in food and animal feed, as a medicine, as a drink, as a dye source, or as a coagulant (Kumar & Sharma, 2020). As for *S. anteuphorbium* is an endemic species belonging to the genus of Senecio from Asteraceae family, which contains a wild variety of secondary metabolites known for their numerous biological properties (Pérez et al., 1999). This plant is largely used in the Moroccan folk medicine to treat several health problems (Saadi et al., 2013).

These plant species are characterized by the presence of several compounds, namely, proteins, polysaccharides, phenol and flavonoids substances, cellulose, and pectin (Madhu et al., 2020; Ouhaddou et al., 2022; Rostami‐Vartooni et al., 2016), which have been proven in the literature to exhibit a coagulant effect allowing their application to remove algal cells and organic matter (El Bouaidi et al., 2022; Graham et al., 2010; Kebaili et al., 2018).

The plant extracts were subjected to an ecotoxicology assessment using four different species considering International Standard Organization (ISO) protocols, namely, *Aliivibrio fischeri*, *Raphidocelis subcapitata*, *Daphnia magna*, and *Sorghum saccharatum*. Subsequently, C/F tests were carried out at different concentrations and pH in order to define the optimal operative conditions.

To the best of our knowledge, this is the first study investigating the coagulation activity and evaluating the ecotoxicological properties of *C. acinaciformis*, *A. americana*, *A. subulate*, and *S. anteuphorbium* the targeted plants or their further application as natural coagulants to pre‐treat contaminated water with CyanoHABs.

## MATERIAL AND METHODS

### Plant material sampling

The fours elected plants, namely, *A. americana*, *C. acinaciformis*, *A. subulate*, and *S. anteuphorbium*, were collected in Morocco (Table 1) and identified according to their morphological and histological characteristics. Samples were preliminary rinsed with tap and distilled water to remove debris and surface‐deposited particles and successively dried at room temperature before their use.

**TABLE 1:** Plant material: Location and sampling date

Species	Harvesting location	Geographic coordinates	Harvesting period
*Agave americana*	Nador area	35.067401	March–April (2019)
		−25.65490	
*Carpobrotus acinaciformis*	Oualidia area	32.730672	March–April (2019)
		−9.049175	
*Austrocylindropuntia subulate*	Tadarte (Ouarzazate area)	32.034360	March–April (2019)
		−9.323058	
*Senecio anteuphorbium*	Souiria laqdima	31.162823	March–April (2019)
		−7.931772	

### Preparation of plant‐based extracts

Plant‐based coagulants were extracted according to the method described by Kukić et al. (2015). All dried plant material was grinded into fine particles and sieved through 0.5 mm sieve. One gram of the sieved powder of each plant material was suspended in 100 mL of a sodium chloride solution (1 M). The obtained suspension was stirred for 10 min in order to extract the coagulating agents and then filtered through 0.45 μm cellulose nitrate membrane filter to remove the insoluble materials. The extracts obtained were stored in a refrigerator at 5°C until use.

### Cyanobacterial culture

The cyanobacterial culture was constituted of *M. aeruginosa* (NCBI accession number: PRJDB11480) sampled during bloom occurrence from the eutrophic reservoir of Lalla Takerkoust, Marrakech (31°36′ N, 8°20′ W), isolated and maintained since October 2015 at a room culture in batch system on Z8 medium (63 μmol photons/m^2^/s with a light/dark of 15/9 h at 26 ± 2°C) and molecularly identified by 16S rRNA sequencing (Tazart et al., 2021).

A cyanobacterial suspension was artificially prepared with *M. aeruginosa* strain to carry out the coagulation tests. Distilled water was inoculated with an exponentially growing *M. aeruginosa* culture (Chen et al., 2015; Sandoval‐Reyes & Ramírez‐Zamora, 2019) in order to obtain a final cell density of 10^6^ cells/mL. This mixture was prepared to stimulate high water turbidity following a cyanobacterial proliferation. *M. aeruginosa* was cultivated in autoclave‐sterilized glass flasks (5 L) to obtain high biomass on liquid Z8 medium for 10 days under the growth conditions mentioned above.

### Ecotoxicology assessment of plant‐based coagulant extracts

After several screening assays, the toxicity assessment of plant‐based coagulants was performed. Extracts were assessed using four different species: (i) *A. fischeri* following the ISO 11348‐3:2007 similarly to Lofrano et al. (2018); (ii) *R. subcapitata* according to ISO 8692:2012 procedure like Libralato et al. (2016); (iii) *D. magna* on the basis of ISO 6341:2012 method; (iv) *S. saccharatum* according to ISO 18763:2016 (Libralato et al., 2016), considering various dilutions (0.1%, 0.4%, 0.7%, 1.25%, 3.12%, 6.25%, 12.5%, and 25%).

Single toxicity data per plant extract and tested dilution were integrated into a final class weight score (0–4) according to Persoone et al. (2003) and Libralato et al. (2010) providing an easy clue of data interpretation from no acute hazard to very high acute hazard.

### Coagulation experiments

The coagulation activity tests were carried out in duplicate using a jar test apparatus equipped with a series of six 500 mL beakers containing the CyanoHABs suspension (10^6^ cells/mL). For each experiment, a defined amount of the coagulating extract was added and mixed at 200 rpm for 2 min at room temperature. The mixing speed was then reduced to 40 rpm for 30 min. Subsequently, the mixture was left to settle for 30 min. Samples from the supernatant were collected, and residual turbidity was measured using turbidimeter. A negative control, which consisted of a suspension of *M. aeruginosa* cells in distilled water without addition of natural coagulants, and a positive control conducted by using aluminum sulfate as coagulant (Al_2_SO_4_, 9H_2_O) were also performed. The effect of pH and concentration on the coagulation activity of each plant‐based extract was studied. The pH value of the CyanoHABs suspension varied from 4 to 10, whereas different coagulant doses were added to achieve 5, 10, 15, 20, 25, and 30 mg/L concentrations. The pH was adjusted to the desired value using either sodium hydroxide (0.1 M NaOH) or hydrochloric acid (0.1 M HCl), similarly to negative and positive controls.

### Statistical analysis

The collected data were statistically analyzed by applying a one‐way and two‐way analysis of variance (ANOVA) analysis. Post hoc differences between group means were evaluated with the Tukey test using SPSS software package (SPSS23). Values of *p* < 0.05 were considered statistically significant. The results were expressed as the mean ± standard deviation. A multivariate analysis was also carried out using principal component analysis (PCA) with XLStat software 2016 version 18.02.01.27444, Addinsoft (France) for screening results (Hakkoum et al., 2021).

## RESULTS

### Toxicity of plant‐based coagulant extracts

**FIGURE 1:**
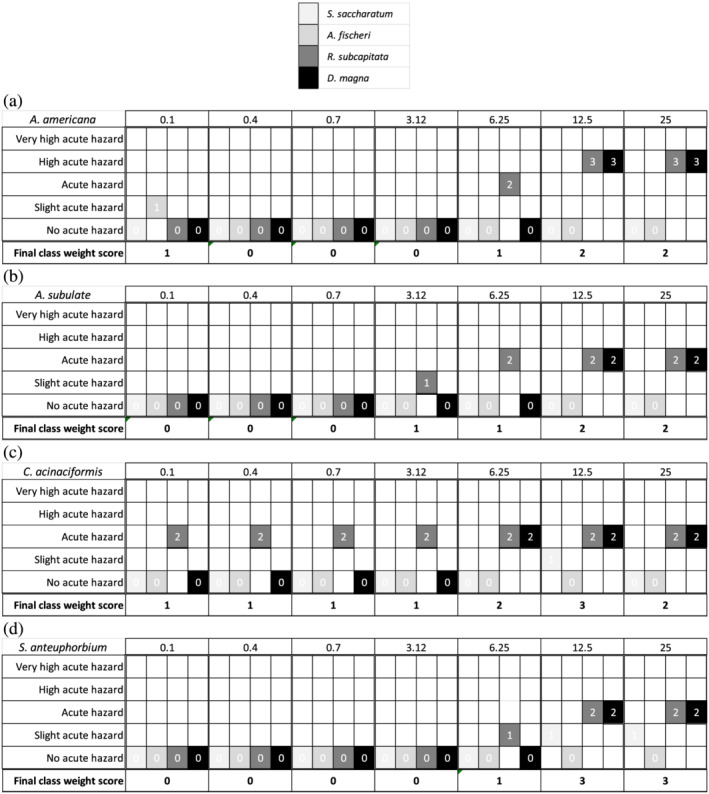
Integrated toxicity results according to Persoone et al. (2003) and Libralato et al. (2010) of the effects on *Agave americana* (a), *Austrocylindropuntia subulate* (b), *Carpobrotus acinaciformis* (c), and *Senecio anteuphorbium* (d) considering the effects of *Sorghum saccharatum* , *Aliivibrio fischeri* , *Raphidocelis subcapitata*, and *Daphnia magna* ; concentrations are in mg/L.

Toxicity results of the plant‐based coagulating extracts on *A. fischeri*, *R. subcapitata*, *D. magna*, and *S. saccharatum* were reported in detail in Figure S1, S2, S3, and S4 for *A. americana*, *A. subulate*, *C. acinaciformis*, and *S. anteuphorbium*, respectively. The integrated toxicity data were reported in Figure 1, including both the classification of toxicity per single tested concentration coupled to the related bioassay and the final class weight score. As a general trend, the 0.1–3.12 mg/L doses presented from no acute hazard (score 0) up to slight acute hazard (score 1). The 12.5 and 25 mg/L doses showed effects from acute hazard (score 2) to high acute hazard (score 3). Extracts from *S. anteuphorbium* were the least toxic (score 0 from 0.1 up to 3.12 mg/L), followed by *A. subulate* (score 0 from 0.1 up to 0.7 mg/L), and *A. americana* (score 0 from 0.4 up to 3.12 mg/L). Extracts from *A. americana* at 0.1 mg/L presented slight acute hazard (score 1) due to the slight acute toxicity showed by *A. fischeri* that disappeared between the subsequent 0.4–3.12 mg/L testing interval. Extracts from *C. acinaciformis* were the most toxic presenting slight acute toxicity (score 1) from 0.1 up to 3.12 mg/L and acute or high acute hazard (score 2–3) from 6.25 up to 25 mg/L.

Thus, the safer green coagulants are *S. anteuphorbium* (0.1–3.12 mg/L) and *A. subulate* (score 0 from 0.1 up to 0.7 mg/L).

### Coagulation

#### Effect of pH and plant‐based coagulants dose on *M. aeruginosa* removal efficiency

**FIGURE 2:**
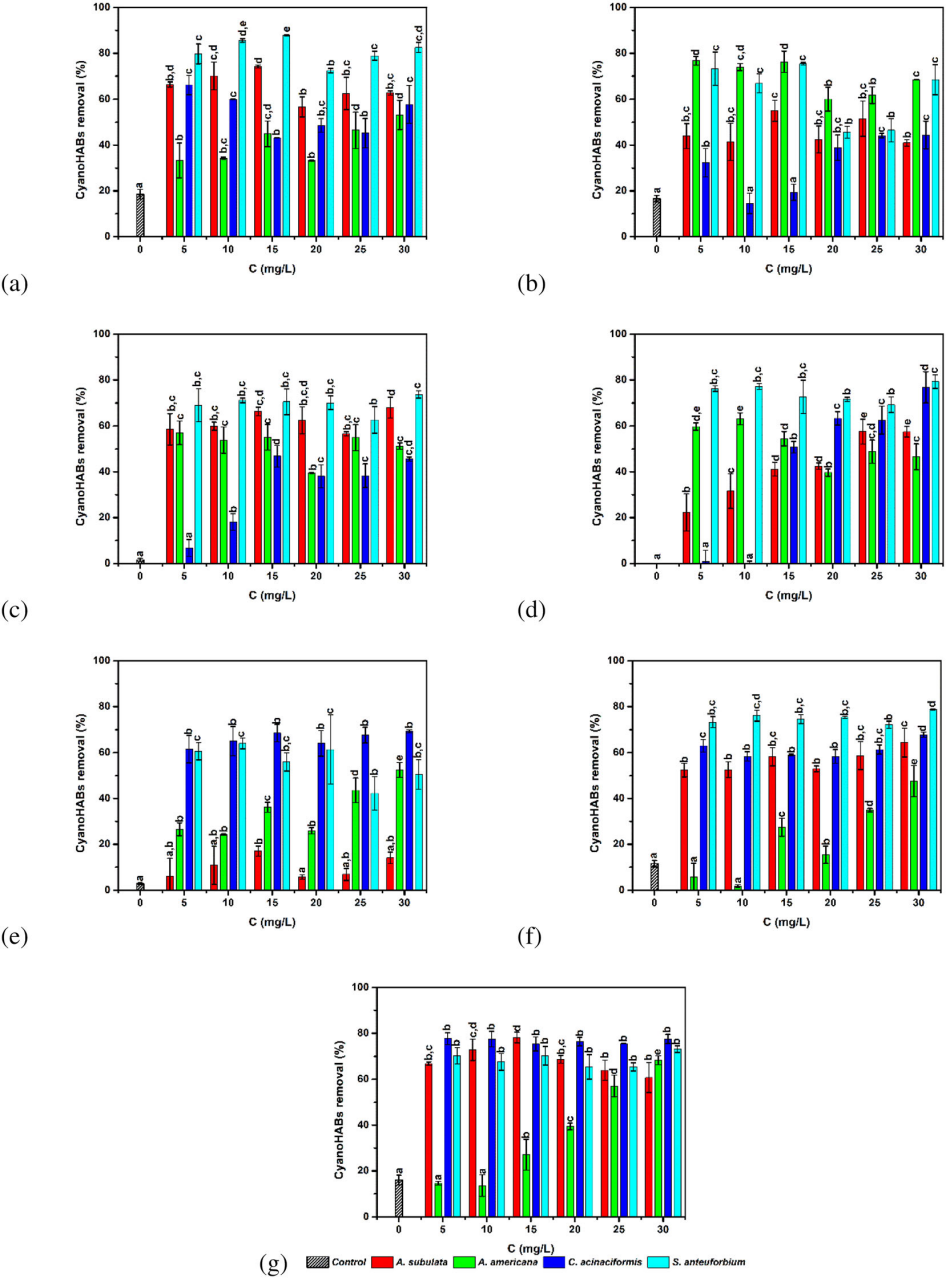
Abatement yield of *Microcystis aeruginosa* cells at different pH and coagulant doses for all studied plant‐based extracts. Results are presented as mean ± SD of three independent assays (letters indicate not significant different data *p* < 0.05 by analysis of variance (ANOVA) and post hoc Tukey's test). (a) pH 4; (b) pH 5; (c) pH 6; (d) pH 7; (e) pH 8; (f) pH 9; (g) pH 10

The efficiency of the four studied plants‐based coagulants to remove *M. aeruginosa* cells was evaluated under pH values ranging from 4 to 10 and coagulant doses from 5 to 30 mg/L in accordance with previous toxicity results. The removal efficiency of CyanoHABs expressed as percentage of turbidity removal as a function of coagulant dose for each pH was shown in Figure 2a–e. As shown, the cyanobacterium abatement varied as a function of pH and coagulant dose differently for all studied plant‐based coagulants. Aluminum salts as positive controls (not reported in Figure 2) showed higher coagulation efficiency (up to 90%) of cyanobacterial cells compared with those obtained by the studied plant‐based coagulants, especially at pH 4, 5, 6, and 10. The removal efficiency was, however, decreased to around 58% at pH 7 using 5, 10, 15, and 20 mg/L of aluminum and to 43% and 48% at pH 8 and 9, respectively. Among all plant‐based coagulants, *S. anteuphorbium* behaviors resulted less dependent from variation of pH and concentrations, and the cyanobacterial removal was different from the control for each experiment. The cyanobacterial removal varied from 72% to 88% at pH 4, from 46% to 75% at pH 5, from 63% to 74% at pH 6, from 69% to 79% at pH 7, from 42% to 64% at pH 8, from 72% to 79% at pH 9, and from 65% to 73% at pH 10. The minimum cyanobacterial removal (42%) was obtained at pH 8 with 25 mg/L, the maximum corresponding to 88% at pH 4 with 15 mg/L. On average, keeping pH constant and varying the concentration of *S. anteuphorbium* extracts, the cyanobacterial removal varied of about 15% at pH 4, 30% at pH 5, 11% at pH 6, 10% at pH 7, 22% at pH 8 and 7% at pH 9, and 8% at pH 10. But for pH 5, the increasing of the extract dose did not produce any statistically significant variation (*p* < 0.05) on cyanobacterial removal for all pH values investigated.

*C. acinaciformis* exhibited the wider range of cyanobacterial removal varying from 43% to 66% at pH 4, from 15% to 44% at pH 5, from 7% to 47% at pH 6, from 0% to 77% at pH 7, from 61% to 69% at pH 8, from 58% to 68% at pH 9, and from 75% to 78% at pH 10. On average, keeping pH constant and varying the concentration of *C. acinaciformis* extracts, the cyanobacterial removal varied of about 23% at pH 4, 30% at pH 5, 40% at pH 6, 77% at pH 7, 8% at pH 8 and 9% at pH 9, and 2% at pH 10. Starting from pH 8, the removal of cyanobacterial was not significantly influenced by the increase of the *C. acinaciformis* extracts concentration. Maximum removal (78%) was obtained at pH 10 with 5 mg/L, but the same result could be achieved at pH 7 by using 30 mg/L.

Also *A. americana* and *A. subulate* extracts behaviors were found strictly dependent from pH and concentrations used. By using *A. americana* extracts, the cyanobacterial removal varied from 33% to 53% at pH 4, from 60% to 77% at pH 5, from 39% to 57% at pH 6, from 40% and 63% at pH 7, from 24% to 52% at pH 8, from 2% to 48% at pH 9, and from 14% up to 68% pH 10. On average, keeping pH constant and varying the concentration of *A. americana* extracts, the cyanobacterial removal varied of 20% at pH 4, 17% at pH 5, 18% at pH 6, 23% at pH 7, 28% at pH 8, 46% at pH 9, and 55% at pH 10. Accordingly, at pH 9 and 10, the cyanobacterial removal statistically significantly (*p* < 0.05) increased by increasing *A. americana* extracts concentrations. By using *A. subulate* extracts, the cyanobacterial removal varied from 57% to 74% at pH 4, from 41% to 55% at pH 5, from 57% to 68% at pH 6, from 22% and 58% at pH 7, from 6 to 17% at pH 8, from 52% to 64% at pH 9, and from 61% to 78% at pH 10. On average, keeping pH constant and varying the concentration of *A. subulate* extracts, the cyanobacterial removal varied of about of 17% at pH 4, 14% at pH 5, 11% at pH 6, 35% at pH 7, 11% at pH 8, 12% at pH 9, and 17% at pH 10. At pH 8, no significant differences were observed in the cyanobacterial removal by *A. subulate* extracts respect to the control. A minimum cyanobacterial removal corresponding to ≈2% and ≈6% were registered at pH 9 with 10 mg/L of *A. Americana* extracts and at pH 8 with 20 mg/L of *A. subulate* extracts, whereas a maximum of 77% and 78% cyanobacterial removal was achieved by using 5 mg/L at pH 5 of *A. americana* extracts and 15 mg/L at pH 10 and L of *A. subulate* extracts.

#### PCAs for screening results

To statistically analyze which parameter has the most significant effect on the behavior of plant‐based coagulants to remove CyanoHABs proliferation, data of pH and coagulant dose experiments were subjected to a PCA analysis.

**FIGURE 3:**
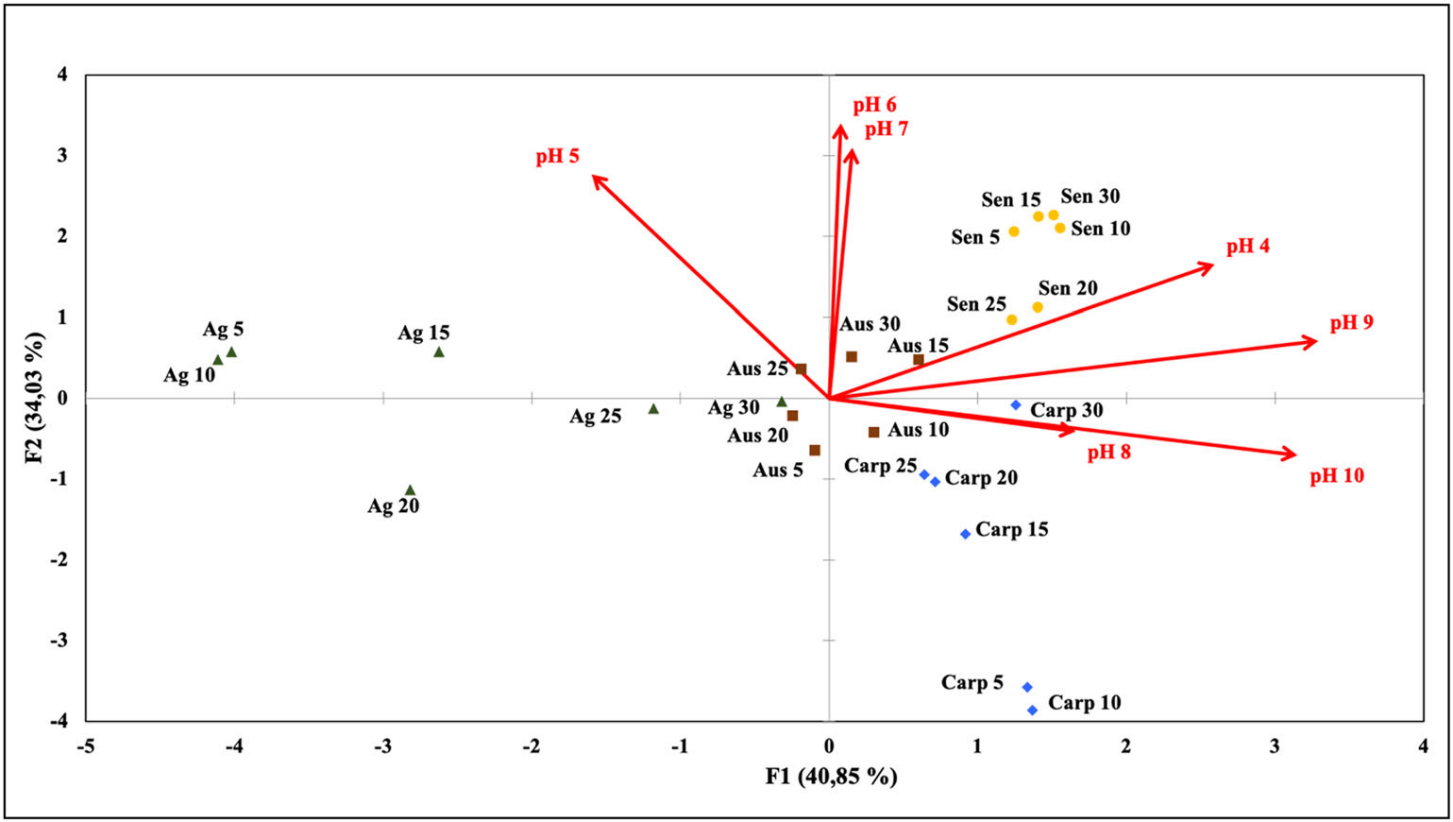
Principal component analyses (PCAs) of plant‐based coagulants studied submitted to different doses and pH. Effect of pH is presented in red. The doses studied are given in several colors according to the plant‐based coagulant tested. Aus, *Austrocylindropuntia subulate*; Carp, *Carpobrotus acinaciformis* ; Ag, *Agave americana* ; Sen, *Senecio anteuphorbium*

The PCA analysis was based on a matrix consisting of three variables namely pH, extract dose, and plant species. The PCA diagram in the bi‐plot form was shown in Figure 3. The variables were mainly correlated with the two axes F1 and F2 in which 75% of the total variance in the data was detected. Indeed, the first axis F1 with 41% of total variance was strongly correlated on the positive side with pH 4, 8, 9, and 10, whereas the second axis F2 with 34% of total variance with the pH 5, 6, and 7. The PCA analysis results allowed distinguishing several groups in relation to the effect of pH for each studied plant‐based coagulant and the corresponding dose for a significant coagulation activity. The first group contains *C. acinaciformis* with a dose of 15, 20, 25, and 30 mg/L, which showed the highest correlation with pH 8, 9, and 10. In the second group containing *S. anteuphorbium*, all the doses were in high correlation with pH 4. The third group includes *A. subulate* with the doses of 5, 10, 15, 20, 25, and 30 mg/L and *A. americana* with the doses of 25 and 30 mg/L. This group has shown no significant relationship between the tested doses and the effect of pH. The fourth group contains *A. americana* with the doses of 5, 10, 15, and 20 mg/L, and finally, the last group includes *C. acinaciformis* with a dose of 5 and 10 mg/L. These groups also revealed no significant relationship between the studied doses and pH.

## DISCUSSION

To the best of our knowledge, no ecotoxicological studies have been previously conducted with the studied plants as natural coagulants. The results obtained indicate that the toxicity may differ significantly depending on the coagulant concentration used to remove cyanobacterial cells from water. However, their level and thus the potential threat of their toxicity in the application in water treatment should not constitute a real danger to living organisms. The tested concentrations of the plant‐based coagulants, ranging from 0.1 to 25%, were selected based on preliminary tests performed to define coagulant doses that may cause a negative or lethal effect on the studied living organisms. The results showed that relatively higher concentrations of the plant‐based coagulants studied, namely, 3.12%, 6.25%, 12.5%, and 25% have exhibited a significant toxic effect on *S. saccharatum*, *R. subcapitata*, *D. magna*, and slightly on *V. fischeri*. This could be related to the biochemical composition of these plants, which may contain compounds that become toxic at high concentrations. Chemical analyses of the studied plants‐based extracts should be performed to determine their composition and their molecular structure. However, while chemical analyses are common for estimating the applicability of various materials, only biological tests can better identify the potential risk of a given material for safety purposes.

The toxic effect of the studied plant‐based coagulants towards the tested organisms varied, and as it can be assumed from the obtained results, low concentrations showed no toxic effect and at the same time showed an important removal efficiency of *M. aeruginosa* cells. Indeed, the literature does not provide any data on the toxicity of the plant‐based coagulants used in this study. However, the response of the toxicity test can be affected by the type of environmental parameters such as specific properties of the sample and specific environmental contamination, which can affect the results.

To the best of our knowledge, this is the first investigation aiming to test the efficiency of *A. americana*, *A. subulate*, *C. acinaciformis*, and *S. anteuphorbium* extracts as natural coagulants to remove CyanoHABs from water. The available literature data are related primarily to the effectiveness of *M. oleifera* in removing *M. aeruginosa* cells (Teixeira et al., 2017) and recently in removing turbidity (Vunain et al., 2019). In this study, the results showed that the four tested plant‐based coagulants presented an interesting efficiency in removing *M. aeruginosa* cells, which are equal to a cyanobacterial bloom. The effectiveness of the four tested plant‐based extracts has been shown to differ significantly depending on the water pH and the coagulant dose used during the coagulation process, which could be comparable with the abatement yields obtained by aluminum sulfate. It was reported that the effect of the pH may depend on the charge of interacting biomolecules present in the plant‐based coagulants (Vishali & Karthikeyan, 2015). The acidic pH was reported to enhance the coagulation efficacy due to the interaction of the coagulant with positively charged ions (Vishali & Karthikeyan, 2015). The same study has also suggested that better coagulation activity could be obtained at neutral pH, which is very economical and convenient to perform the treatment at real pH. Katalo et al. (2018) demonstrated that *M. oleifera* could be effectively applied to reduce turbidity with no need to adjust the pH of the contaminated water as compared with aluminum coagulant. It has been reported that over the normal pH range of water (6–8), particles almost always carry a negative surface charge and thus are colloidally stable and resistant to aggregation. In this case, coagulants are needed to stabilize the particles by adsorbing counter ions to neutralize the particle charge. This could explain the effect of basic pH and coagulant dose on the coagulation efficiency of *A. americana*. According to several studies, *A. americana* contains a large proportion of proteins, amino acids, pectin, lignin, and hemicellulose that have different surface charges depending on pH (Madhu et al., 2020; Schabort et al., 1978). It has been established that proteins have negative charges at alkaline pH and positive charges at acidic pH, while some of the amino acids can provide positive or negative charges depending on the type of amino acid involved in the coagulation process. Similarly, it was stated that *A. subulate* contains protein, fiber, fatty acids, and pectin (Lucio & Alexandra, 2009). It can be deduced that *A. americana* and *A. subulate* have cationic surface charges under neutral and acidic pH, which could explain the efficiency of removal obtained under this pH through strengthened charge neutralization coagulation mechanism (Ang & Mohammad, 2020). Similarly, Li et al. (2015) showed that cationic coagulant showed excellent removal of negatively charged particles while performing poorly in the removal of positively charged suspension. Moreover, the same was observed on chitosan, which contains a high content of amino groups. It provides a cationic charge at acidic pH, supports the destabilization of colloidal suspension, and promotes large flocs and subsequently a rapid‐settling (Nechita, 2017). According to several studies, natural coagulants with positive charges can coagulate negatively charged particulate and colloidal materials via adsorption and hydrophobic flocculation (Karbassi & Heidari, 2015; Roussy et al., 2005; Saranya et al., 2014). *C. acinaciformis* presents a high content of phenolic groups such as hydroxyl and carboxyl ones (Rostami‐Vartooni et al., 2016). It can be assumed that this plant‐based coagulant has an anionic surface identical to that of *M. aeruginosa* cells at neutral pH. The coagulation efficiency obtained varies at certain doses as a function of pH and might be explained by the bridging mechanism, in which the coagulant and the particle carry the same surface charges, which involves the adsorption of coagulant segments on adjacent colloidal surfaces, thus linking them each other (Bolto & Gregory, 2007). It has been reported that *S. anteuphorbium* contains a major component on hydrocarbons, cellulose followed by alcohols, ketones, and aldehydes (Ouhaddou et al., 2022). According to the literature, hydrocarbons and some cellulose derivatives are characterized as nonionic polymers. These non‐ionic polymers rely on hydrophobic interactions to bring the particles together, and while pH does not affect the speciation of the nonionic polymer, it does alter the speciation of the particle itself. Thus, higher pH decreases the effectiveness of non‐ionic polymers because *M. aeruginosa* particles tend to become more hydrophilic at higher pH as the proportion of negative charges increases (Dayarathne et al., 2021). In this context, it is widely known that biological coagulants can perform such role having particular bio‐macromolecular structures with a wide range of functional groups that can interact with contaminants (Crini, 2005; Sharma et al., 2006; Swati & Govindan, 2005). For this reason, each tested plant showed a different efficiency removal according to the pH used and the mechanism induced in the process of coagulation activity. Several mechanisms of coagulation were proposed to explain the destabilization of colloids and suspensions by added coagulants, namely, polymers bridging, polymer adsorption, precipitative coagulation, charge neutralization, and depletion flocculation (Bolto & Gregory, 2007; Bratby, 2006; Jiang, 2001; Stechemesser, 2005).

Furthermore, the effectiveness of plant‐based coagulants also depends on the dose of the used coagulant; thus, insufficient coagulant amounts or overdosing would results in a poorer coagulation performance (Choy et al., 2015; Hassan et al., 2009). In this study, the highest CyanoHABs removal efficiency was observed at the doses of 5 mg/L for *C. acinaciformis* and *A. americana* and15 mg/L for *A. subulate* and *S. anteuphorbium*, presenting no toxicity or slight acute toxicity level. However, for certain plant‐based coagulants, smaller doses of 5 mg/L could also be used, because no significant enhancement has been observed when using high amounts of coagulants. Kukić et al. (2015) demonstrated that the best coagulation activity was obtained at smaller doses of 0.125 and 0.25 mL/L of *V. faba* extracts with removal efficiencies of stock kaolin suspension as synthetic turbid water of 52% and 54%, respectively. The positive effect achieved with the smaller or the higher doses of coagulant could be due to the amount of the active components and their structure, in relation to several factors including the initial concentration of cyanobacterial cells, the initial turbidity, the water pH, the stirring‐speed, and the time of sedimentation. Nevertheless, the plant‐based coagulants discussed in this work are promising biological coagulants for environmental preservation and purification, also from an economical point of view. The main advantages related to natural coagulants derived from plant as compared with chemical coagulants are their sustainability and their availability, which probably makes them economically less expensive (sludge handling and coagulant cost) (Saleem & Bachmann, 2019). Despite the fact that the performance and benefits of the aforementioned natural coagulants have been proven in lab‐ and/or bench scale, there is some reluctance regarding their cost‐effectiveness and consistency of performance in actual treatment processes.

Limited information on the cost of raw natural coagulants is available (Ang & Mohammad, 2020; El Bouaidi et al., 2022). In some studies, the cost of natural coagulants has been shown to be less than that of chemical coagulants generally requiring intensive pH adjustment of the water to be treated to achieve the desired removal efficiency (Choy et al., 2014). In contrast, various studies have revealed that the use of natural coagulants requires additional costs dedicated to their extraction, storage, transportation, and handling, and sometimes more dosage are needed to be reach the desired efficiency in comparison with chemical coagulants (Katalo et al., 2018; Megersa et al., 2016). In some cases, certain conventional coagulants may be less expensive; for example, alginate is more costly than aluminum salts, but it is used in smaller quantities, making it less expensive than alum salts (Bixler & Porse, 2011; Çoruh, 2005). To overcome these uncertainties, depth economical investigations should be carried out to evaluate the perception towards natural coagulants in DWTPs.

In view of the preceding considerations, it can be deduced that the plant‐based coagulants studied can be used as an alternative to conventional coagulants, taking into consideration the optimal pH and dose for each DWTPs.

## CONCLUSIONS

Coagulation effectiveness was evaluated in removing cyanobacterial cells (*M. aeruginosa*) by testing coagulating extracts derived from new plants materials, namely, *A. subulate*, *C. acinaciformis*, *A. americana*, and *S. anteuphorbium*, as alternatives to chemical coagulants. Results from the screening and the PCA analyses showed that the studied plants could be effectively used to remove *M. aeruginosa* cells depending on the pH of water and the used coagulant dosage. *A. americana* at doses of 5, 10, 15, 20, 25, and 30 mg/L, *A. subulate* at doses of 5, 10, 15, 20, 25, and 30 mg/L, and *C. acinaciformis* at doses of 5 and 10 mg/L showed high cyanobacterial cells removal efficiency with no effect depending on the pH of water. Nevertheless, *C. acinaciformis* have evidenced effective removal with doses of 15, 20, 25, and 30 mg/L at pH 8, 9, and 10, and *S. anteuphorbium* have shown a removal efficiency at all studied doses with a high effect of pH 4. The integrated toxicity data carried out on the specific living organisms *S. saccharatum*, *A. fischeri*, *R. subcapitata*, and *D. magna* suggested that concentrations below 3.12 mg/L can be considered as safe, while below 12.5 mg/L can present slight acute hazard on a case‐by‐case basis.

Nature based coagulants can provide an interesting alternative to chemical coagulants in drinking water treatment, but operational cost, local circular economies, and carbon footprint must be further analyzed.

## CONFLICT OF INTEREST

The authors declare no conflict of interest.

## Supporting information


**Figure S1.** Toxicity effects on *A. fischeri* , *R. subcapitata,* *D. magna* , and *S. saccharatum* to *A. americana* extracts; concentrations are in mg/L; **, *** indicate p < 0.01 and p < 0.001 between samples by ANOVA.
**Figure S2.** Toxicity effects on *A. fischeri* , *R. subcapitata,* *D. magna* , and *S. saccharatum* to *A. subulate* extracts; concentrations are in mg/L; *, **, *** indicate p < 0.05, p < 0.01, and p < 0.001 between samples by ANOVA.
**Figure S3.** Toxicity effects on *A. fischeri* , *R. subcapitata,* *D. magna* , and *S. saccharatum* to *C. acinaciformis* extracts; concentrations are in mg/L; *, **, *** indicate p < 0.05, p < 0.01, and p < 0.001 between samples by ANOVA.
**Figure S4.** Toxicity effects on *A. fischeri* , *R. subcapitata,* *D. magna* , and *S. saccharatum* to *S. anteuphorbium* extracts; concentrations are in mg/L; *, **, *** indicate p < 0.05, p < 0.01, and p < 0.001 between samples by ANOVA.

## Data Availability

The data that support the findings of this study are available from the corresponding author upon reasonable request.
