# Supplementary material for: Nature‐based coagulants for drinking water treatment: An ecotoxicological overview
Source: Water Environ Res. 2022 Aug 27;94(8):e10782. doi: 10.1002/wer.10782 (PMC9545364; doi:10.1002/wer.10782)

**Supplementary Materials**

Toxicity results of the plant-based coagulating extracts on *A. fischeri*, *R. subcapitata,* *D. magna*, and *S. saccharatum* were reported in detail in Figure 1S, 2S, 3S, and 4S for *A. americana, A. subulate, C. acinaciformis,* and *S. anteuphorbium*, respectively.

The bioluminescence of *A. fischeri* in presence of *A. americana* showed a slight inhibition with about 14% as maximum inhibition at the highest dose (25 mg/L). The inhibition percentages were observed to be -3% to 14 % at coagulant doses of 3.12 mg/L and 25 mg/L, respectively. The results of the toxicity test with *A. subulate,* showed that the fluorescence of *A. fischeri* was not inhibited. The percentage values were ranging from -7% to -21% in the doses of 6.25 mg/L and 0.1 mg/L respectively. Regarding the toxicity of *C. acinaformis* extracts, the inhibition of the fluorescence of *A. fischeri* varied from -65% to 18% in the doses of 0.4 mg/L to 12.5 mg/L respectively. For *S. anteuphorbium*, the results showed values ranging from -42% to 7% for coagulant doses between 0.4 mg/L and 25 mg/L, respectively. As a general outcome, the tested plant-based coagulating extracts have no significant toxic effects on the bioluminescence of *A. fischeri*.

The results of the toxicity assessment tests performed with *R. subcapitata* in the presence of plant-based coagulating extracts showed that the 0.1, 0.4 and 0.7 mg/L doses are presenting a biostimulation effect on *R. subcapitata* cells growth for all tested plants, except for *C. acinaciformis* extract, which shows inhibition of algal growth of 76%, 68%, and 82% respectively. Biostimulation results suggested potentiality for the extract to act as a eutrophication agent. Conversely, 3.12, 6.25, 12.5 and 25 mg/L of extracts showed inhibition of algal cell growth in all the plant-based coagulants.

Toxicity test with *D. magna* evidenced no specific changes in the percentages of living daphnids after exposure to the plant-based coagulating extracts at low concentrations of 0.1, 0.4, 0.7, and 3.12 mg/L. The immobilization was observed at high concentration of 6.25, 12,5 and 25 mg/L in all plant-based coagulant extracts tested up to 100%.

According to Libralato et al. (2016a) results, *S. saccharatum* germination index (I, %) data were clustered into three main groups: (i) no effect (80% ≤ GI ≤ 120 %); (ii) biostimulation (GI > 120 %); and (iii) inhibition (GI < 80 %). The analysis of the toxicity data of *A. americana* revealed that the tests performed with *S. saccharatum* pointed out an inhibition of GI at doses of 3.12, 6.25, 12.5 and 25 mg/L. However, no evident toxicity effect has been observed at low concentrations of 0.1, 0.4, 0.7 and 1.25 mg/L. Furthermore, the GI observed at 0.1, 0.4, and 0.7 mg/L showing value >120%, indication biostimulation in *S. saccharatum*. Regarding *A. subulate*, the data analysis of *S. saccharatum* showed that 3.12, 6.25, and 12.5 mg/L presented a quite complete absence of ecotoxicity. In contrast, the 25 mg/L dose showed a very high toxicity with a GI of 20%. However, the 0.1, 0.4 and 0.7% doses were found to provide a biostimulation effect on *S. saccharatum* growth, with GI > 120%.

For *C. acinaformis* extracts, the results of *S. saccharatum* tests showed that 0.1, 0.7, 3.12 and 6.25 mg/L doses present no toxicity effect. Nevertheless, 12.5 and 25 mg/L doses inhibited the growth of *S. saccharatum*, where the GI takes values of 21% and 54% respectively. However, only the dose of 0.4 mg/L evidenced the presence of biostimulation of *S. saccharatum* growth (GI = 142%). As for *S. anteuphorbium* extracts, the test results showed that almost all the doses studied present a biostimulation effect (GI > 120%) on *S. saccharatum* growth, except for 12.5 and 25 mg/L doses, which showed an inhibition effect of 76 and 25%, respectively.

Figure 1S Toxicity effects on *A. fischeri*, *R. subcapitata,* *D. magna*, and *S. saccharatum* to *A. americana* extracts; concentrations are in mg/L; **, *** indicate p < 0.01 and p < 0.001 between samples by ANOVA.


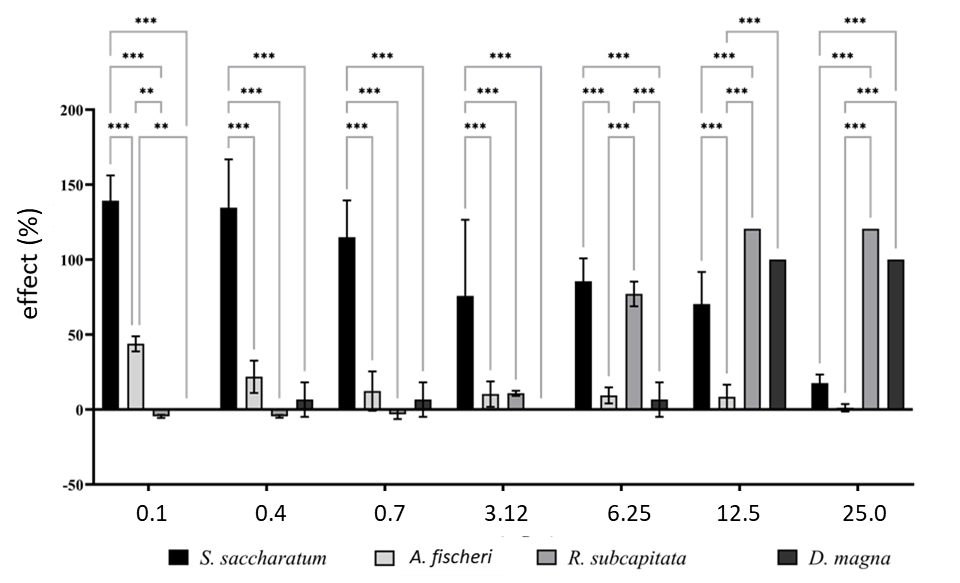


Figure 2S Toxicity effects on *A. fischeri*, *R. subcapitata,* *D. magna*, and *S. saccharatum* to *A. subulate* extracts; concentrations are in mg/L; *, **, *** indicate p < 0.05, p < 0.01, and p < 0.001 between samples by ANOVA.


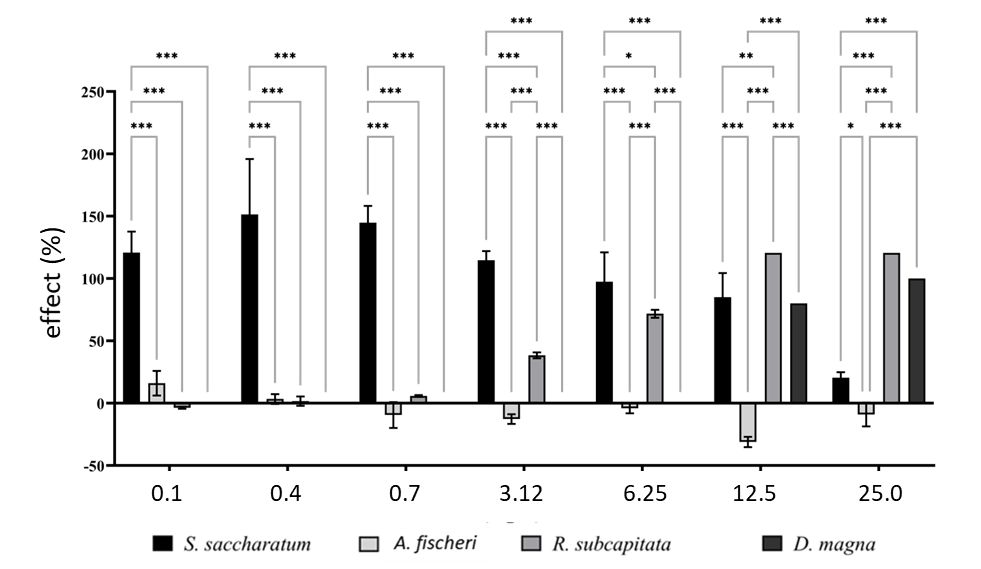


Figure 3S Toxicity effects on *A. fischeri*, *R. subcapitata,* *D. magna*, and *S. saccharatum* to *C. acinaciformis* extracts; concentrations are in mg/L; *, **, *** indicate p < 0.05, p < 0.01, and p < 0.001 between samples by ANOVA.


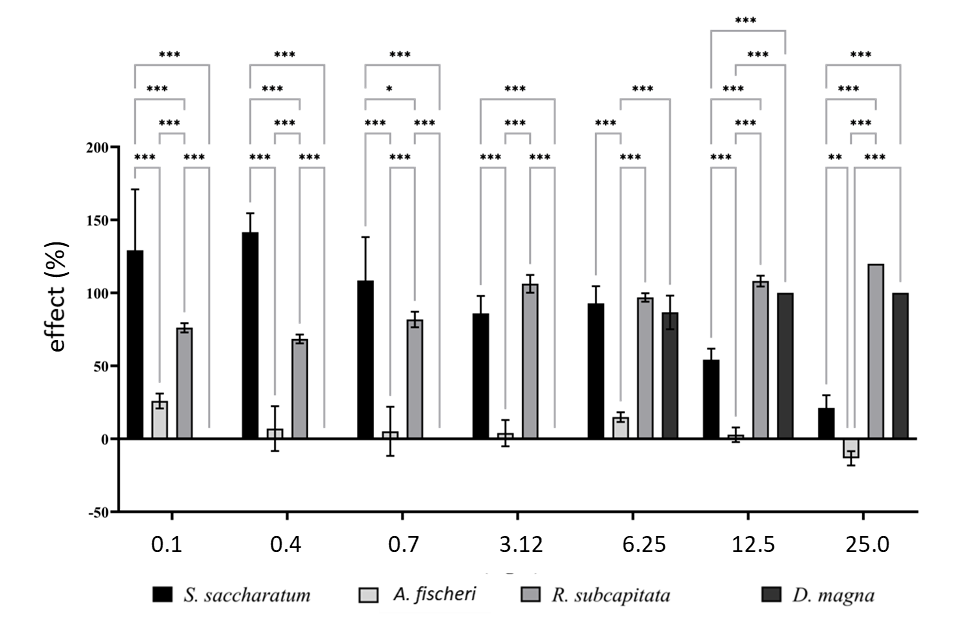


Figure 4S Toxicity effects on *A. fischeri*, *R. subcapitata,* *D. magna*, and *S. saccharatum* to *S. anteuphorbium* extracts; concentrations are in mg/L; *, **, *** indicate p < 0.05, p < 0.01, and p < 0.001 between samples by ANOVA.


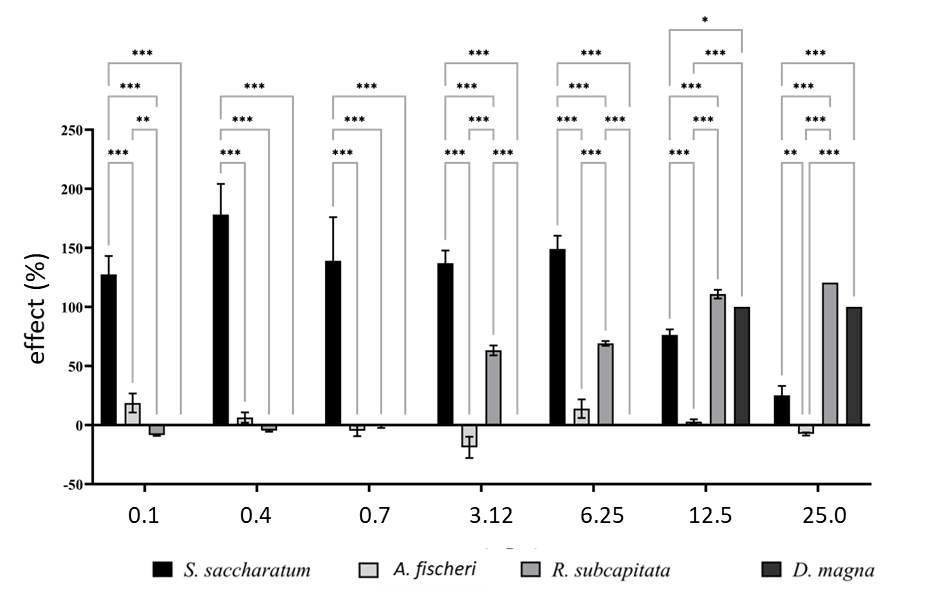

Supplement: Supplementary file 1 — Figure S1. Toxicity effects on A. fischeri , R. subcapitata, D. magna , and S. saccharatum to A. americana extracts; concentrations are in mg/L; **, *** indicate p < 0.01 and p < 0.001 between samples by ANOVA. Figure S2. Toxicity effects on A. fischeri , R. subcapitata, D. magna , and S. saccharatum to A. subulate extracts; concentrations are in mg/L; *, **, *** indicate p < 0.05, p < 0.01, and p < 0.001 between samples by ANOVA. Figure S3. Toxicity effects on A. fischeri , R. subcapitata, D. magna , and S. saccharatum to C. acinaciformis extracts; concentrations are in mg/L; *, **, *** indicate p < 0.05, p < 0.01, and p < 0.001 between samples by ANOVA. Figure S4. Toxicity effects on A. fischeri , R. subcapitata, D. magna , and S. saccharatum to S. anteuphorbium extracts; concentrations are in mg/L; *, **, *** indicate p < 0.05, p < 0.01, and p < 0.001 between samples by ANOVA. [file WER-94-0-s001.docx]
